# Crowdsourced benchmarking of taxonomic metagenome profilers: lessons learned from the sbv IMPROVER Microbiomics challenge

**DOI:** 10.1186/s12864-022-08803-2

**Published:** 2022-08-30

**Authors:** Carine Poussin, Lusine Khachatryan, Nicolas Sierro, Vijay Kumar Narsapuram, Fernando Meyer, Vinay Kaikala, Vandna Chawla, Usha Muppirala, Sunil Kumar, Vincenzo Belcastro, James N. D. Battey, Elena Scotti, Stéphanie Boué, Alice C. McHardy, Manuel C. Peitsch, Nikolai V. Ivanov, Julia Hoeng

**Affiliations:** 1 PMI R&D, Philip Morris Products S.A., Quai Jeanrenaud 5, 2000 Neuchâtel, Switzerland; 2 Data Science and Informatics, Corteva Agrisciences, Ascendas IT Park, Madhapur, Hyderabad, 500081 India; 3 Department of Computational Biology of Infection Research, Helmholtz Centre for Infection Research (HZI), Braunschweig, Germany; 4 Member of the Scoring Review Panel for the Microbiomics Challenge, Neuchâtel, Switzerland

**Keywords:** Crowdsourcing, Computational method benchmarking, Metagenomics, Taxonomic profiling, Microbiome, Bacterial communities, Sbv IMPROVER

## Abstract

**Background:**

Selection of optimal computational strategies for analyzing metagenomics data is a decisive step in determining the microbial composition of a sample, and this procedure is complex because of the numerous tools currently available. The aim of this research was to summarize the results of crowdsourced sbv IMPROVER Microbiomics Challenge designed to evaluate the performance of off-the-shelf metagenomics software as well as to investigate the robustness of these results by the extended post-challenge analysis. In total 21 off-the-shelf taxonomic metagenome profiling pipelines were benchmarked for their capacity to identify the microbiome composition at various taxon levels across 104 shotgun metagenomics datasets of bacterial genomes (representative of various microbiome samples) from public databases. Performance was determined by comparing predicted taxonomy profiles with the gold standard.

**Results:**

Most taxonomic profilers performed homogeneously well at the phylum level but generated intermediate and heterogeneous scores at the genus and species levels, respectively. kmer-based pipelines using Kraken with and without Bracken or using CLARK-S performed best overall, but they exhibited lower precision than the two marker-gene-based methods MetaPhlAn and mOTU. Filtering out the 1% least abundance species—which were not reliably predicted—helped increase the performance of most profilers by increasing precision but at the cost of recall. However, the use of adaptive filtering thresholds determined from the sample’s Shannon index increased the performance of most kmer-based profilers while mitigating the tradeoff between precision and recall.

**Conclusions:**

kmer-based metagenomic pipelines using Kraken/Bracken or CLARK-S performed most robustly across a large variety of microbiome datasets. Removing non-reliably predicted low-abundance species by using diversity-dependent adaptive filtering thresholds further enhanced the performance of these tools. This work demonstrates the applicability of computational pipelines for accurately determining taxonomic profiles in clinical and environmental contexts and exemplifies the power of crowdsourcing for unbiased evaluation.

**Supplementary Information:**

The online version contains supplementary material available at 10.1186/s12864-022-08803-2.

## Background

Microorganisms, mainly bacteria, archaea, fungi, and viruses, colonize almost every part of the terrestrial biosphere—soil, water, and living organisms. In humans, microbes cover all external and internal epithelial surfaces, such as the skin, oral sphere, airways, genitals, and digestive tract [1], and these microbes generally live in symbiosis with their host, contributing to host homeostasis by supporting metabolic functions, assisting in the development of immunity, protecting against pathogens, and regulating physiological functions or behaviors through the gut–brain axis [2, 3]. Factors such as genetics, diet, age, antibiotic use, toxins, and toxicants can influence and can consequently perturb the composition and thus the function of microbiota [1], introducing an imbalance, which is termed “dysbiosis.” This state is associated with several medical conditions and changes in the composition of a microbiome may be used as biomarkers (fingerprints) of disease [1]. Therefore, accurate determination of the composition of a microbiome is the starting point for gaining a further understanding its functions and causalities, such as host–microbiome and intra-microbiome interactions and possibly their link to diseases. Such research opens up a novel therapeutic development landscape as well as various application opportunities in diagnostics [4, 5].

By applying modern high-throughput sequencing technologies to a biological sample, it is possible to obtain its genomic snapshot. The microbial composition of the sample can be estimated by computational assignment of sequencing reads to microbial taxa and quantifying their abundance. Numerous computational methods and software tools have been developed for taxonomic profiling from 16S or shotgun sequencing data [6], but limited information on the performance and applicability of these computational methods makes it difficult to choose the most appropriate strategy. Evaluation of published computational methods is generally limited to developers benchmarking their method against other existing methods [7]. However, this non-blinded evaluation is often restricted and challenging because of the number and choice of methods to be compared. Such self-assessment may also lead to biased results [7] and, consequently, a low consensus on benchmarking datasets and evaluation metrics in metagenomics.

The sbv IMPROVER (systems biology verification for Industrial Methodology Process Verification) crowdsourcing project aims to verify methods and data in systems biology [8]. sbv IMPROVER has shown its usefulness in benchmarking computational methods to address scientific questions articulated in crowdsourced challenges involving systems toxicology, species translatability, and diagnostic signature identification [9–14]. The sbv IMPROVER Microbiomics Challenge was designed to assess the performance of off-the-shelf metagenomics software data analysis pipelines as a whole—that is, from quality control to taxonomy profiling of relative abundance and taxonomic assignment of bacterial communities—rather than assessing individual steps of the process. This new challenge falls within the continuum of crowdsourced initiatives such as the Critical Assessment of Metagenome Interpretation (CAMI) challenge (http://www.cami-challenge.org/), which have evaluated methods in metagenomics for assembly, binning, and taxonomy profiling [15]. However, unlike in similar studies, the benchmarking datasets selected for this study cover a broad set of features (e.g., habitat, host origin, dataset complexity, sequencing technique, and dataset construction method) and thus allow broader and more detailed assessment of the prediction quality of the selected profilers.

Participants in the Microbiomics Challenge were provided shotgun reads generated either by sequencing commercially available DNA isolated from a mixture of selected microorganisms with known relative abundances or by simulating reads in silico by using complete bacterial genomes from the National Center for Biotechnology Information (NCBI) GenBank database. To investigate the impact of microbial composition complexity and biases on the performance of computational methods for metagenomics taxonomy profiling, we simulated microbiome samples with higher numbers of species and incorporated (or not) a biased representation of AT- or GC-rich bacterial species. The participants were asked to predict, at the phylum, genus, and species levels, the composition of bacterial communities in each sample on the basis of their relative abundance. The participants had the freedom to use any private or public dataset to set up and test their approach. After completion of the challenge, anonymized participant predictions were scored using predefined complementary binary classification and abundance metrics for performance assessment and identification of the best-performing approaches. This manuscript summarizes the results and lessons learned from the Microbiomics Challenge and extended benchmarking post-analyses, including additional taxonomic metagenome profiler pipelines and simulated and real metagenomics datasets representative of microbiomes from various environmental settings and human organs.

## Results and discussion

### The metagenomics pipeline using Kraken combined with Bracken performed best on the challenge dataset

**Fig. 1:**
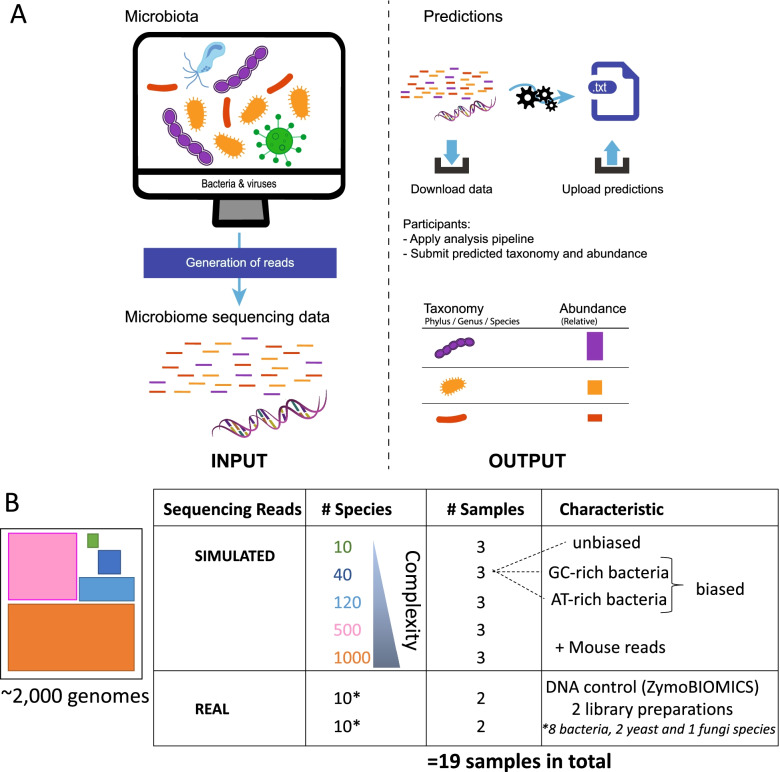
Overview of the objective and dataset of the Microbiomics Challenge. Schematic description of the challenge (**A**). Participants were provided simulated metagenomics datasets representative of samples with increasing bacterial composition complexities and biases and including mouse host-read contamination. Real metagenomics datasets were generated from the sequencing of two independent libraries prepared from the commercially available ZymoBIOMICS DNA standard extracted from a known mixture of microorganisms, including eight bacterial species and two yeasts (**B**)

**Fig. 2:**
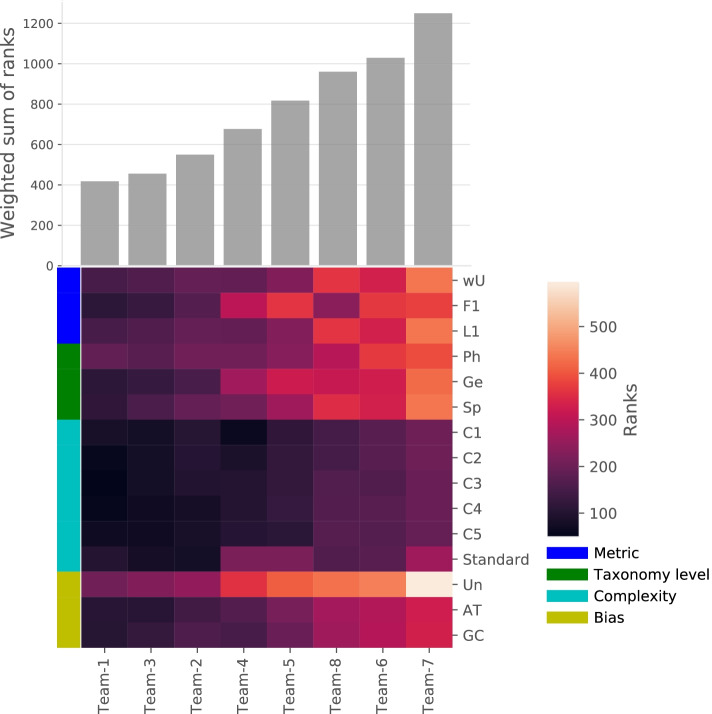
Final team ranking in the sbv IMPROVER Microbiomics Challenge. Bar plot of the weighted sum of ranks (wsr) sorted from the lowest (best) to the highest (worst) wsr. A heatmap shows the wsr stratified by metrics (wU, weighted UniFrac; F1, F1 score; L1, L1 norm), taxonomic levels (Ph, phylum; Ge, genus; Sp, species), complexity (C; Standard corresponds to the real ZymoBIOMICS DNA standard), and sequence bias status (Un, unbiased sample; AT or GC, AT/GC-rich biased samples)

The challenge participants’ mission was to apply a taxonomic metagenome profiling pipeline to simulated and real shotgun metagenomics sequencing samples of various compositions and complexities to predict the taxa present in each sample and their relative abundance at the phylum, genus, and species levels (Fig. 1, with panel A showing the competition schematic representation and panel B illustrating the strategy for competition metagenomics datasets generation). To raise awareness about this new challenge, we have presented the Microbiomics Challenge in conferences, organized a webinar, and described it in detail on the sbv IMPROVER website. Overall, seven worldwide teams participated in the challenge and submitted a total of eight predictions on the sbv IMPROVER platform. The scorers received the anonymized submissions after the challenge closed. The scoring process evaluated how well, in comparison with the gold standard (Additional file 1), a method predicted the presence or absence of taxa by using the F1 score as a binary metric and rebuilt the relative abundances by using the L1 norm and weighted UniFrac as abundance metrics. For each submission, the scores were converted to ranks, which were aggregated in the form of a weighted sum of ranks (wsr). The taxonomic metagenome profilers used by the participants included kaiju, CLARK, and Kraken combined or not with Bracken (Fig. 2 and Table 1). The pipelines combined these tools with read preprocessing and filtering steps and used the full or restricted contents of the microbial genome databases. The pipeline of Team 8 did not use classical tools but instead combined read alignment to genomes by using blastn and their assignment to taxa by using TargetMiner and the RDP database. The submission with the lowest overall wsr of 418 won the challenge (Fig. 2 and Additional file 2, Sheet A). The best-performing pipeline used the taxonomic metagenome profiler Kraken in combination with Bracken, while the second and third best pipelines included Kraken without Bracken. The difference between the second and third teams was the use of bacteria-only and full-content databases, respectively. To assess how far the predictions were from randomness, the participants’ prediction scores were compared with distributions of random prediction scores stratified by taxonomic level, unbiased or AT−/GC-rich-biased bacterial composition, and sample complexity (Additional file 3). Team submissions with scores considered to be random increased with higher sample complexities, and the F1 scores were more affected than the L1 norm and weighted UniFrac scores. This latter observation indicates that binary (presence/absence) assignment of microorganisms to specific taxonomic communities had more impact on the teams’ performance than the prediction of abundances. Predictions at the phylum level were more accurate than predictions at the species level, indicating that accurate qualitative and quantitative identification of bacterial communities at a deeper taxonomic level is more challenging. These results are consistent with previous CAMI reports of a notable decrease in performance below the family level [15]. The increased variability of the teams’ scores at lower taxonomic levels illustrates the emergence of differences in pipeline performance, which may be negligible at the phylum level. Each team’s pipeline exhibited small differences in performance in the presence and absence of AT−/GC-rich biases. This suggests that the workflows were not influenced by this factor, which is in contradiction with previous findings showing that GC bias has an effect in next-generation sequencing data, for example, on genome assembly [16] or that the bias introduced by a certain proportion of AT−/GC-rich bacteria was insufficiently pronounced to lead to a substantial effect (Additional file 3).

**Table 1:** Taxonomic metagenome profiling pipelines used by participants in the challenge

Team	Preprocessing	QC	Filtering	Classification	Quantification
1	NA	FastQC (version not specified)	NA	Kraken v1.0 against bacteria	Bracken (version used for the challenge, commited code: March 5, 2018)
2	NA	FastQC (version not specified)	FastQC	Kraken v1.0 using bacterial database	Kraken v1.0
3	NA	FastQC (version not specified)	FastQC	Kraken v1.0 against full database	Kraken v1.0
4	Removal of host contamination by BWA v0.7.12 mapping on mouse genome	BWA v0.7.12 mapping against complete NCBI bacterial genomes	SAMtools v1.8 filtering in to keep bacterial reads only	BWA v0.7.12 mapping against complete NCBI bacterial genomes	Kaiju v1.6.2
5	NA	BWA v0.7.12 mapping against RefSeq bacterial genomes	SAMtools v1.8 filtering in to keep bacterial reads only	BWA v0.7.12 mapping against RefSeq bacterial genomes	Kaiju v1.6.2
6	PEAR v0.9.10 for merging of overlapping read pairs	FastQC v0.11.7	NA	CLARK v1.2.5 using NCBI/RefSeq bacterial and archaeal genomes as reference (13.06.2018)	CLARK v1.2.5
7	NA	FastQC (version not specified)	NA	Kaiju v1.6.2 using NCBI/RefSeq bacterial and archaeal genomes as reference	Kaiju v1.6.2
8	Selection of 16S rRNA reads by BLASTN (version not specified) in clustered RDP database and concatenation of read pairs	SeqTools (version not specified)	NA	BLASTN (version not specified) against concatenated read pairs (using RDP as reference database) followed by taxon determination using TargetMine custom script	Sequence counts divided by 16S copy number

**Fig. 3:**
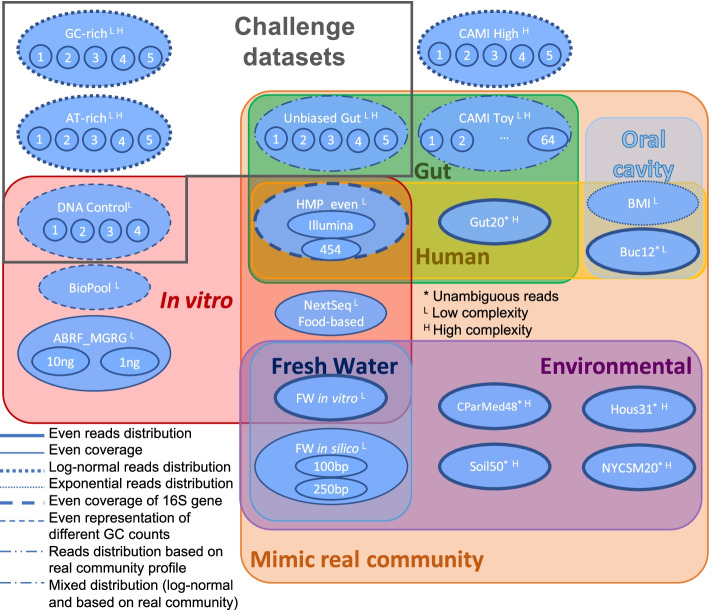
Overview of the shotgun metagenomics datasets used for the challenge and extended benchmarking analysis. A total of 104 real and simulated metagenomics shotgun datasets grouped into 19 categories (surrounded by ovals) representative of microbiome samples from various environmental settings and mammalian organs were generated for the challenge or selected from previous studies for the extended benchmarking analysis. The characteristics of the simulated datasets are indicated in the legend. The line of the oval represents the mixing model used for creating each benchmarking dataset in the group. Dataset properties are shown by the background color shading. Datasets with Shannon index values below and above a threshold value of 3 were considered as having low (L) and high (H) complexity, respectively

**Fig. 4:**
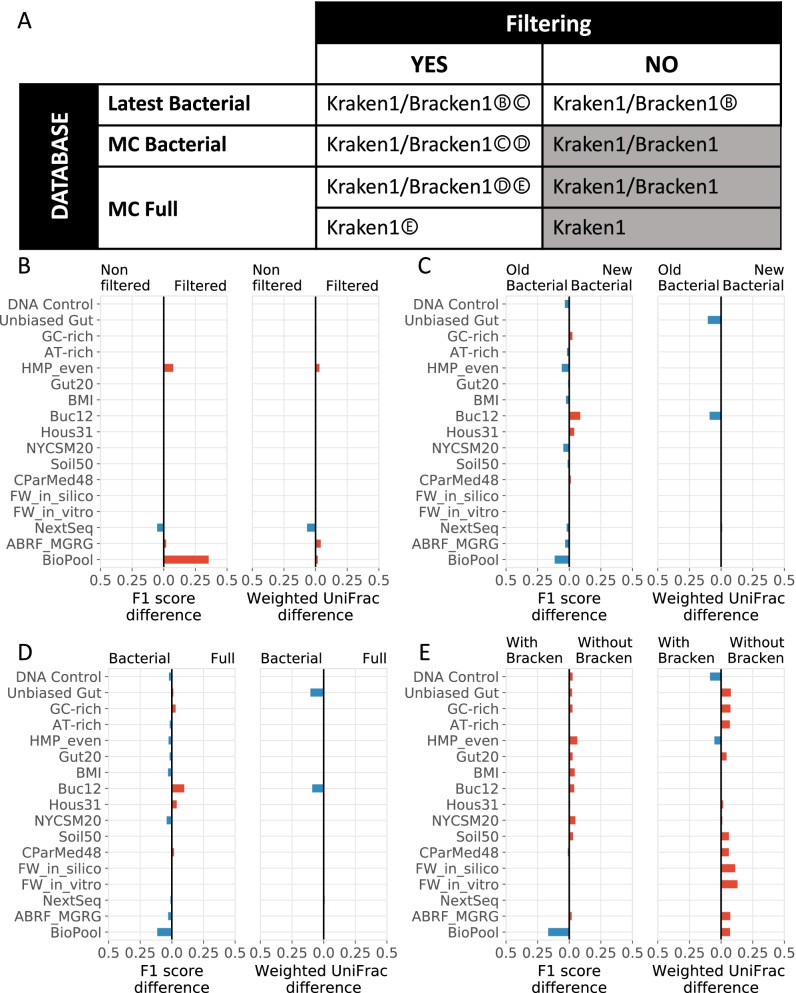
Impact of various parameters on the performance of Kraken. Schematic representation of the sets of factor combinations for investigating their impact on the performance of the Kraken tool, which was used in the three best-performing pipelines. Combinations shaded in grey were not investigated (**A**). The impact of quality control read filtering (**B**), database version (**C**), database completeness (**D**), and count estimate by using Bracken (**E**) were evaluated. The absolute difference in F1 scores or weighted UniFrac scores between two options (option 1 on the left and option 2 on the right side for each diverging bar chart) were calculated for each dataset for the factors investigated. The color of the bars illustrates whether the option 1 (blue) or option 2 (red) had a larger score

To better understand the difference in performance of the top three pipelines using the Kraken tool, we investigated the impact of factors such as read filtering, bacterial genome database content associated with versions and completeness, and read count estimation by using Bracken. To ensure that the results and conclusions were not specific to the challenge dataset, we expanded the analysis to additional publicly available simulated and real benchmarking metagenomics datasets representative of various microbiome compositions from various environmental settings and human organs (Fig. 3 and Additional file 2, Sheet B). The analysis was conducted by comparing the performance of the winning pipeline with various factor combinations (Fig. 4 [Panel A] and Additional file 2, Sheet C). The results were expressed as the difference in the F1 scores and weighted UniFrac scores for the factor under evaluation (Fig. 4 [Panels B–E] and Additional file 2, Sheet D). In general, we did not observe large absolute differences in scores (i.e., exceeding 0.25 for both weighted UniFrac and F1 scores) for any of the factor combinations. Except for the NextSeq dataset, the read filtering (Fig. 4 [Panel B]) generally exhibited a beneficial effect for the in vitro datasets and no effect on the scores for the simulated datasets. The in vitro datasets are real sequencing data that contain low-quality reads and contaminants such as adapters, which are not necessarily present in simulated datasets, and should be filtered out to increase the quality of the read mapping and of the assignment to and quantification of taxa. With the exception of the Buc12 and Hous31 datasets, using an older version of a bacterial genome database surprisingly resulted in better predictions for the presence/absence of taxa than can be obtained with a more recent database version and, in general, did not impact the estimated species abundances (Fig. 4 [Panel C]). Similar results were obtained when comparing the impact of the reference database contents. The database restricted to bacterial sequences resulted in overall better scores than those obtained when using the full database of bacteria, viruses, and archaea (Fig. 4 [Panel D]).

Collectively, these results lead to the following conclusions. The use of more recent and complete microbial genome databases with Kraken offers new informative genomic content that improves species identification in datasets for which the information was missing. However, the more recent databases may increase the number of false positives by possibly increasing non-specific read mapping on microbial genomes newly available in the database. These observations may also be linked to the benchmarking datasets themselves, which are mostly simulated data predominantly using bacterial genomes available in older databases at the time they were generated, highlighting the limitations of using in silico data.

Contrary to our expectations, Bracken negatively impacted the scores obtained with Kraken for taxon quantification for most of the datasets, with the exception of the DNA Control and HMP_even datasets, for which beneficial effects were observed (Fig. 4 [Panel E]). Further analysis indicated that the Kraken default pipeline rounds the taxon abundances to two decimals and therefore eliminates taxa with an abundance less than 0.005 from the final report. When Bracken is combined with Kraken for re-estimating taxon abundances, it uses a complete non-rounded Kraken output report directly. Thus, after the Bracken-based re-estimation, taxa excluded as noise by the Kraken default parameters may appear in the final profile, resulting in a higher number of false-positive counts.

### Extended benchmarking of taxonomic metagenome profilers confirmed Kraken combined with Bracken as the overall best-performing pipeline across various microbiome composition sample datasets

**Fig. 5:**
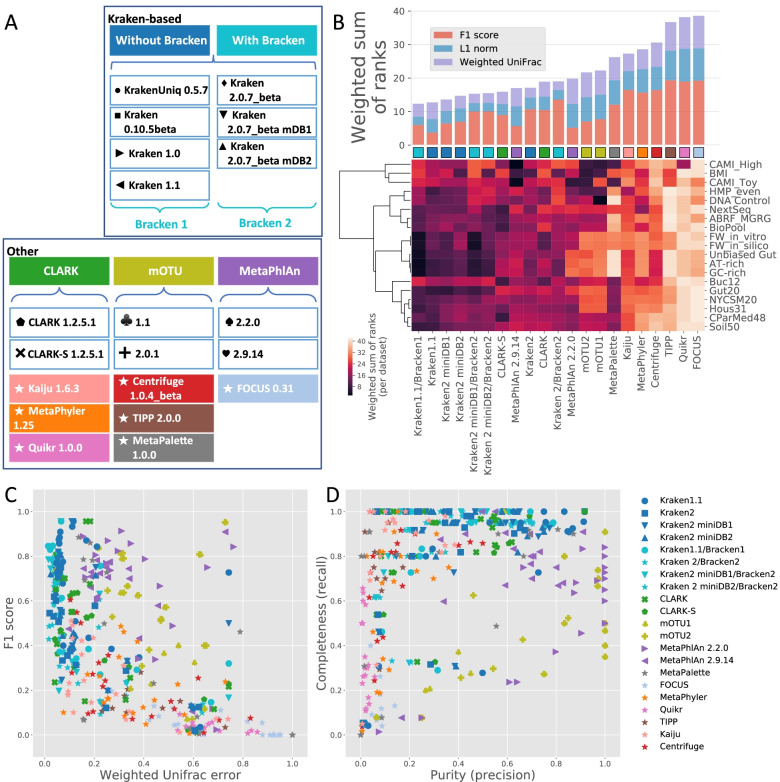
Extended benchmarking analysis of metagenomics taxonomy profiler pipelines across various datasets. Collection of benchmarked taxonomic profilers (**A**). Bar plot showing the weighted sum of ranks (wsr) of scores calculated by using three metrics: F1 score, L1 norm, and weighted UniFrac. Colors in the bars highlight the contribution of each metric to the final wsr. Taxonomic profiling pipelines are sorted from the lowest (best) to the highest (worst) wsr. The heatmap represents the wsr obtained for each taxonomic profiler per group of benchmarking datasets (**B**). Scatter plots of weighted UniFrac scores versus F1 scores (**C**) or purity (precision) versus completeness (recall) (**D**) for each benchmarked taxonomic profiler and dataset group. Each dot corresponds to the mean of scores obtained for a group of sample datasets. The color and shape of each dot are associated with a taxonomic profiler pipeline

In order to extend the conclusions of this challenge and evaluate their robustness, we expanded the benchmarking analysis to a total of 21 taxonomic metagenome profiling pipelines (Fig. 5 [Panel A] and Additional file 2, Sheet E) to predict the composition of 104 metagenomic samples, which were combined into 19 dataset groups to account for the compositional bias of some sample types (Fig. 3 and Additional file 2, Sheet B). This extended analysis focused on benchmarking pipelines at the species level only, given the more substantial differences in performance observed at this taxonomic level. The benchmarked tools included those used for the challenge for deeper investigation of their performance on extended benchmarking datasets. Other tools were also selected if they were frequently benchmarked in recent publications (from 2015), open source, still maintained, and covered various algorithm types. Although several papers have already reported informative results on benchmarking taxonomic metagenome profiler tools [15, 17–24], the present investigation is, to our knowledge, the broadest benchmarking analysis to date, as it evaluated multiple tool/dataset combinations. We also investigated the performance of the tools with default parameters, excluding any sort of dataset-related pipeline optimization. The additional benchmarking datasets were selected from previous publications as representative of various microbiome compositions from differing environmental settings and human/mouse organs and covering various abundance distributions and complexity levels. Because of the need for gold standards for tool performance evaluation, most of these datasets were simulated, which remains a limitation for benchmarking metagenomics tools. Therefore, we also included real datasets from the sequencing of microbiome samples constituted in vitro by mixing multiple microorganismal (bacterial and yeast) strains in predefined proportions. Although synthetic in their composition, these datasets include artifacts such as biases, sequence errors, and contamination (to some extent) generated during the sample processing (e.g., DNA extraction and library preparation) and high-throughput sequencing in the laboratory. Since each of the tested profilers had its own reference database, we processed the output profiles and gold standards using one reference NCBI-based taxonomy tree (Additional file 4 and Additional file 5) for a fair comparison. This required translation of the taxIDs associated with each predicted taxonomy profiles into taxIDs associated with this reference NCBI-based taxonomy tree.

Figure 5 shows that the in various versions and pipeline combinations with and without Bracken, the Kraken tool outperformed the other tools in many cases across all datasets (Additional file 2, Sheet F). However, the eight best-performing pipelines, which also included CLARK-S and MetaPhlAn 2.9.14, did not show large differences in wsr values, which ranged between 13 and 18. This result indicates that the overall performance may not vary substantially among these pipelines, although differences did emerge when investigating the wsr stratified by metrics. The pipeline that combined Kraken 1.1 with Bracken 1 performed the best, supplanting Kraken version 2. When focusing on the challenge dataset only, the pipeline that used Kraken 1.1 with Bracken 1 was also the highest performer among all benchmarked tools (Additional file 6). Hence, the larger benchmarking confirms the challenge results, and the benchmarking of tools across multiple datasets ensured that the results and conclusions were not dataset-specific. With the exception of the CAMI and BMI datasets, Kraken pipelines with and without Bracken predicted species abundances accurately (Fig. 5 [Panel C]), with 75% of the sample datasets falling within a range of weighted UniFrac distance values of 0–0.22. In contrast, the F1 scores exhibited high variability, with 75% of sample datasets scattered between 0.32 and 1, possibly explained by the high variability in precision (75% of samples within a range of 0.21–1), while the recall values were greater than 0.82 for 75% of the sample datasets (Fig. 5 [Panel D]). In comparison with the Kraken pipelines, MetaPhlAn exhibited less variability in the F1 scores (> 0.61 for 75% of sample datasets) but rather scattered weighted UniFrac values (75% of samples within a range of 0–0.44) (Fig. 5 [Panel C]). CLARK-S was the second best-performing tool after Kraken, with similar score behaviors. Tools such as FOCUS, Quikr, and TIPP exhibited the poorest performance in both qualitative and quantitative species estimates across all sample datasets (Fig. 5 [Panel B]).

These results indicate that Kraken and CLARK-S kmer-based pipelines showed a better overall performance in qualitative and quantitative profiling at the species level than marker-gene-based pipelines (MetaPhlAn and mOTU) and other pipelines. These findings are consistent with those of previous benchmarking analyses [17] that provided similar conclusions at the phylum and genus levels [25] as well as a recent meta-analysis of several benchmarking studies that highlighted the consistent top ranking of CLARK, Kraken, and One Codex [18]. The impact of the default reference databases used by the tools may also account for the observed differences in the prediction performance of the taxonomy metagenome profilers. However, this aspect was not evaluated, per se, in this work, as the scope was to evaluate off-the-shelf software tools provided with their default parameter options and built-in reference databases. Strict benchmarking of algorithm performances would necessitate the use of an identical set of microbial genome references, which is not always easily achievable, as certain taxonomic metagenome profiler tools come with precomputed reference databases. The LEMMI benchmarking platform permits continuous integration of taxonomic profilers and binners and enables the use of an identical set of references for comparing tool performance [26]. Our results also highlight the variable and lower performance of Kraken (depending on pipeline version) and CLARK-S kmer-based pipelines compared with that of MetaPhlAn and mOTU marker-gene-based pipelines in predicting species presence/absence, with the former suffering from a higher level of false-positive predictions. The use of reference databases packaged with the software tools and/or the differing read-mapping strategies may explain this observation. A possible increase in non-specific mapping to reference genomes for kmer-based approaches—less likely in the context of a restricted list of marker genes—may contribute to these false positives. In contrast, Kraken and CLARK-S performed better in estimating species abundances than the marker-gene-based and other pipelines.

### Determining an adapted stepwise- and context-dependent threshold for filtering out low-abundance species is key to increasing the performance of most profiling pipelines

**Fig. 6:**
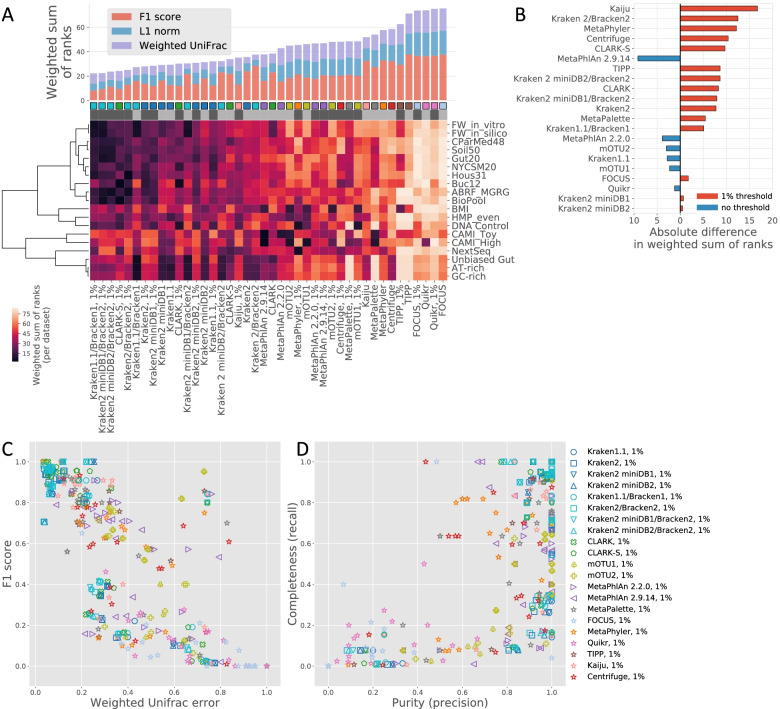
Impact of 1% filtering threshold for predicted lowest-abundance species on the performance of benchmarked profilers. Bar plot showing weighted sum of ranks (wsr) of scores without and with filtering out of the 1% least abundant species. Colors in the bars highlight the contribution of each metric to the final wsr. Taxonomic profiling pipelines are sorted from the lowest (best) to the highest (worst) wsr. The heatmap represents the wsr obtained with and without filtering out of the 1% least abundant species for each taxonomic profiler per group of benchmarking datasets (**A**). Bar plot showing the difference in wsr obtained with and without filtering out of the 1% least abundant species. The color and orientation of the bars illustrate the directionality of the difference (**B**). Scatter plots of weighted UniFrac scores versus F1 scores (**C**) or purity (precision) versus completeness (recall) (**D**) for each benchmarked taxonomic profiler and dataset group. Each dot corresponds to the mean of scores obtained for a group of sample datasets. The color and shape of each dot are associated with a taxonomic profiler pipeline

Previous works have reported a reduction in the number of false positives after filtering out low-abundance species from taxonomy profiles [19, 20, 24]. Therefore, we investigated the impact of removing low-abundance species by using an arbitrary abundance cutoff of 1% [20] (Fig. 6, Additional file 7, and Additional file 2, Sheet F). With the exception of MetaPhlAn and mOTU, both marker-gene-based taxonomic profilers, almost all tools exhibited a systematic improvement in performance across sample datasets when low-abundance species were filtered out, indicating that most tools may not reliably assess low-abundance species (Fig. 6 [Panels A and B]). The filtering step most benefited Kaiju, Kraken 2/Bracken 2, and Metaphyler in terms of wsr, while tools such as FOCUS and Kraken 2 miniDB1/miniDB2 were the least affected. Kraken2 can be used with any database or with preconstructed databases such as MiniKraken2_v1_8GB, an 8-GB Kraken 2 database constructed from the RefSeq bacteria, archaea, and viral libraries, or MiniKraken2_v2_8GB, an 8-GB Kraken 2 database constructed from the RefSeq bacteria, archaea, and viral libraries and the GRCh38 human genome assembly. The filtering out of low-abundance species translated into an overall increase in F1 scores, mostly for samples with lower complexity, explained by the greater precision (Fig. 6 [Panels C and D] and Additional file 7) due to a decrease in false positives, but at the cost of recall in some cases, reflected by an increase in false negatives. In addition, the number of true positives decreased in sample datasets such as CAMI and simulated challenge datasets for which samples from low to high complexities were aggregated, leading to an overall decrease in the F1 score. In contrast, filtering low-abundance species did not markedly change the weighted UniFrac values, indicating that filtering low-abundance species affects qualitative rather than quantitative predictions by improving the tradeoff between false-positive and false-negative classifications (Additional file 7).

**Fig. 7:**
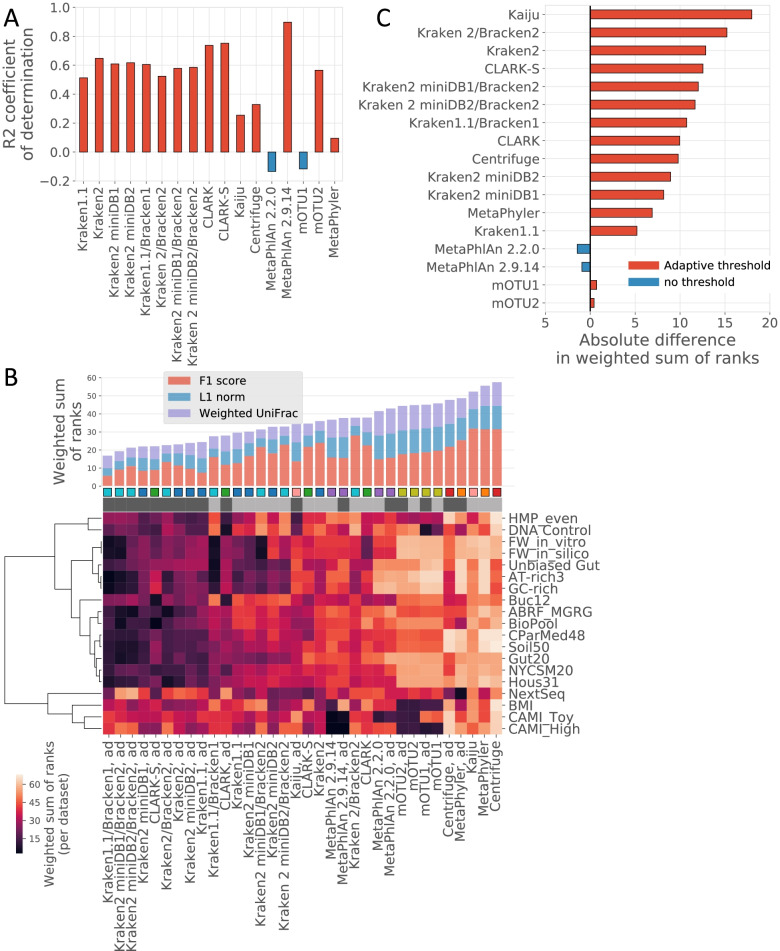
Impact of filtering out predicted low-abundance taxa using context-dependent adaptive thresholds on taxonomic profilers’ performance. The correlation between Shannon indices calculated from benchmarking datasets, gold standards, and the outputs of each tool (**A**). Bar plot showing the wsr of scores calculated by using three metrics (F1 score, L1 norm, and weighted UniFrac) without and with filtering out of low-abundance species by using context-dependent adaptive thresholds. Colors in the bars highlight the contribution of each metric to the final wsr. Taxonomic profiling pipelines are sorted from the lowest (best) to the highest (worst) wsr. The heatmap represents the wsr obtained without and with filtering out of low-abundance species by using context-dependent adaptive thresholds for each taxonomic profiler per group of benchmarking datasets (**B**). Bar plot showing the difference in wsr obtained without and with filtering out of low-abundance species by using context-dependent adaptive thresholds. The color and orientation of the bar illustrate the directionality of the difference (**C**)

Every biological sample contains a long tail of low-abundance species, which might present a challenge for classifiers. However, in some cases, these species represent the majority of a sample’s contents. Thus, applying a fixed threshold to remove false-positive classifications might, in fact, eliminates vast proportion of variability in samples. To investigate whether a more informed filtering threshold than 1% could help optimize the tradeoff between false-positive and true-positive species (Fig. 7 and Additional file 2, Sheet F), we studied the relationship among species abundance levels, false-positive/true-positive tradeoff, and within-sample diversity using the Shannon index. The false positives consisted mainly of predicted low-abundance species (Additional file 8). A clear separation was observed between true positives and false positives for most tools, with the exception of MetaPhlAn and mOTU. No clear relationship was observed between the Shannon diversity index and abundant species when all sample dataset types were plotted. However, we observed that stratification of gut and non-gut sample datasets revealed an inverse linear relationship between the Shannon index and species abundances in non-gut sample datasets (Additional file 8). We cannot exclude that this may have been linked to the intrinsic properties of those datasets (i.e., specific read distribution) or to real gut and non-gut microbiome structures. For non-gut sample datasets, it may be necessary to develop an adapted filtering threshold by using a linear or other function between the Shannon diversity index and species abundances. However, we used a more exploratory and empirical approach to define and investigate optimal low-abundance species filtering thresholds for gut and non-gut datasets. To achieve this, we first verified that the Shannon indices calculated from predicted taxonomy profiles correctly replicated those computed from the gold standards (Additional file 9). With the exception of MetaPhlAn 2.2.0, mOTU1, and MetaPhyler, which yielded coefficients of determination close to 0, the tools predicted microbial diversities accurately, with MetaPhlAn 2.9.14 and CLARK pipelines outperforming other pipelines with R^2^ values > 0.7 (Fig. 7 [Panel A]). Next, we tested a stepwise adaptive filtering approach based on Shannon index ranges empirically defined as follows: for non-gut sample datasets, the filtering thresholds were 1, 0.1%, and 0 for Shannon index ranges of 0–2.5, 2.5–4.5, and > 4.5, respectively; for gut sample datasets, the filtering thresholds were 0.1% and 0 for Shannon index ranges of 0–2.5 and > 2.5, respectively (Additional file 10). Although the effect on MetaPhlAn and mOTU was limited, the adaptive filtering benefited all pipelines tested, showing a clear and positive effect on precision and limited negative impact on recall in comparison with the observations made when a 1% filtering threshold was applied (Fig. 7 [Panels B and C] and Additional file 7).

Overall, these results indicate that the presence/absence of low-abundance species was not reliably predicted by most benchmarked pipelines. Filtering out species with abundances lower than 1% helped improve the overall performance, although at the cost of more species being missed. Predicted species diversity, reflected by the Shannon index, was generally well replicated by most pipelines and correlated with species abundances in the non-gut sample datasets. Defining stepwise and context-dependent Shannon index-based filtering thresholds empirically was shown to be a better approach than not filtering, using a 1% or 0.1% filtering threshold, and it helped improve the performance of all pipelines except MetaPhlAn. After filtering out low-abundance species, the Kraken 1.1/Bracken 1 pipeline remained the best-performing pipeline, closely followed by other versions of Kraken and Bracken in combination and CLARK-S.

## Conclusions

Numerous computational approaches have been proposed for determining microbial composition from shotgun metagenomics sequencing data. However, the accuracy of predicting the presence/absence of taxa and their abundances varies among the tools and depends on the microbiome context.

Previous attempts to evaluate metagenome classifiers were performed using various types of in silico and in vitro simulated communities [15, 17–24]. However, the quality of classification was usually assessed using the data from certain habitats, with fixed read length/simulation model and/or organism abundance distribution within the benchmarking datasets. Therefore, even metagenomics experts may encounter difficulties in selecting the most appropriate off-the-shelf tools for research because of the lack of systematic, broad, and independent comparative analyses. To support decision-making, the crowdsourced sbv IMPROVER Microbiomics Challenge and extended analyses benchmarked—to our knowledge— the largest set of off-the-shelf pipelines applied on more than 100 real and simulated shotgun metagenomics datasets. These sample datasets were representative of microbiomes from various environmental settings and human organs, with low to high complexities, and biased or unbiased for GC- or AT-rich bacterial genomes. The results showed that a pipeline combining Kraken with Bracken performed the best in predicting and quantifying the presence/absence and abundances of species—which is more challenging than predicting the higher taxonomic levels—across 104 datasets grouped into 19 categories. CLARK-S, another kmer*-*based approach, also performed well. Most taxonomic metagenome profiling pipelines were not reliable in predicting the presence/absence of low-abundance species; with the exception of marker-gene-based pipelines such as MetaPhlAn and mOTU, the profiler performance increased when low-abundance species were filtered out. This can be explained by the following hypothesis: marker-gene-based tools (MetaPhlAn and mOTU) use for classification a well-maintained, and usually curated, reference database with reference genes highly specific to a particular taxon. Thus, the risk of incorrect assignments when classifying reads in metagenomic sample is relatively low. At the same time, such reference databases are less comprehensive, which increases the number of false-negative predictions, especially when analyzing metagenomics samples from poorly studied environments. Conversely, tools such as Kraken, CLARK, Kaiju, or Centrifuge that use larger, non-curated reference databases for read classification have a higher chance of taxonomic misassignment owing to the large number of sequences that are indistinguishable between different taxa. However, they produce a lower false-negative rate because of their better representation of all species in the reference database. Nevertheless, establishing a cutoff threshold is not a trivial task because, for many metagenomes, low-abundance species represent the main contents of the sample. However, in our study, determination of adaptive filtering thresholds from information on sample diversity appeared to be a better approach for improving precision without affecting recall than using a constant filtering threshold such as 1% or 0.1%. This finding opens avenues for further investigations and development of robust methods or improvement of current taxonomic profilers for inferring appropriate filtering thresholds. Overall, this work provides lessons on the performance and applicability of computational pipelines optimally depending on the microbiome context for the accurate determination of taxonomic profiles. Such accurate taxonomic profiling is a critical step for supporting future developments in environmental and clinical research, for example with the discovery of metagenomic-based biomarkers associated with specific disease states such as ulcerative colitis and Crohn disease. By leveraging the power of crowdsourcing as well as extended post-challenge collaborative benchmarking work among the organizing team, scoring review panel members, and best performers, this study has highlighted the importance of unbiased and independent evaluation of computational methods to achieve more generalizable results and confidence in scientific conclusions in metagenomics.

## Material and methods

### Goals and rules of the challenge

The sbv IMPROVER Metagenomics Challenge (November 2017 to June 2018) aimed to evaluate the performance of computational metagenomic analysis pipelines for their ability to accurately recover the relative abundance of microbial communities at the phylum, genus, and species levels of the taxonomy tree.

### Generation of metagenomics datasets

Nineteen samples were simulated in silico or generated by sequencing the DNA from samples of known bacterial composition. Minimum information on the generation of the dataset was released to the participants during the challenge. A copy (14.07.2017) of the NCBI’s bacterial taxonomy tree and genomes and chromosomes (complete genome sequences of 1886 reference species) were downloaded and frozen as the “reference dataset” for generating and analyzing metagenomics datasets and later for evaluating the participants’ prediction submissions. The data were described as originating from mouse microbiome samples.

### Simulated metagenomics sequence dataset

Sequencing reads for 15 of the 19 samples (Fig. 1, Additional file 11, and Additional file 2, Sheet G) were generated computationally by using the reference dataset and ART simulation tools [27], with the parameters set to simulate next-generation sequencing reads from an Illumina HiSeq4000 sequencer (2 × 150-bp paired-end reads). Reads from sequencing mouse cecal samples were mapped onto the mouse host genome (m38) and matching reads were retained and added to the simulated reads as sequence-read contaminants, representing 8–11% of the total reads of a sample. Simulated samples with increasing complexity (number of species) were generated in the presence or absence of AT- and GC-rich sequence biases (Additional file 2, Sheet G).

To create unbiased samples with low and medium complexity (fewer than 500 species), the number of reads were fixed at 1, 5, and 9 million (+ 0 to 10%) for samples 19, 11 and 7, respectively (Additional file 2, Sheet G). The species were randomly (uniform distribution) drawn from a list of candidate species identified from mouse gut microbiomes. The number of reads to be generated per sample reflected the relative species abundance identified in the gut microbiomes.

To create unbiased samples with higher complexity (500 or more species), the coverage for each species was drawn from a log-normal distribution with a mean of − 1 and a standard deviation of 1 in accordance with the CAMI publication’s recommendations [15]. The first 50% of the targeted number of species were randomly selected from a uniform distribution of species among the reference species. The remaining 50% of species were obtained by randomly selecting genera that contained 4–11 sequenced species and subsequently selecting all the sequenced species of each genus until the targeted number of species was reached or exceeded This would allow us to evaluate the capability of the computational analysis pipelines to discriminate bacteria at the species level.

To create AT- and CG-rich biased samples with low, medium, and high complexities, the coverage for each species was taken from a log-normal distribution with a mean of − 1 and standard deviation of 1; for samples with fewer than 500 species, a mean of 1.2 and standard deviation of 0.8 were used, thus ensuring a smooth coverage gradient from high to low complexity. AT- and GC-rich species were characterized by an AT-to-GC ratio greater than 60%. The samples included 50% of non-biased sequencing reads and 50% of sequencing reads biased towards AT- or GC-rich species. Among the 50% of non-biased sequencing reads, half were generated by randomly selecting species among references genomes by using uniform distribution, and the second half were obtained by selecting closely related (same genera) species as described above. Among the remaining 50% of sequencing reads biased towards AT- or GC-rich species, half were generated by randomly selecting species among reference genomes from AT- or GC-rich species by using uniform distribution; the second half were obtained by selecting closely related (same genera) AT- or GC-rich species as described above.

### Real metagenomics sequencing dataset

The four remaining samples were derived from the commercially available ZymoBIOMICS™ Microbial Community DNA Standards and corresponded to mixtures of genomic DNA extracted from pure cultures of eight bacteria and two yeasts at known ratios. The GC content of the genomes covered a range of 32.7–66.2%. These samples from two library preparations were sequenced in a 2 × 151-bp paired-end run on an Illumina HiSeq4000 sequencer.

### Data preparation and release

#### Data files

Sequencing data were provided in the form of two FASTQ files per sample (paired end). The complete dataset could be retrieved by downloading a tar archive, which was split in four files and included gzipped FASTQ files for the 19 samples.

#### Prediction submission requirements

The participants were asked to predict the taxonomic composition of the provided samples at the phylum, genus, and species levels. The taxonomic composition was to be expressed as the relative abundance (percentage) of each taxon in the sample’s microbiome. The participants were required to ensure that the percentages given for each taxon from the same rank summed up to less than or exactly 100. Submissions had to comply with the Bioboxes profiling format (https://github.com/bioboxes/rfc/blob/master/data-format/profiling.mkd).

The information on the taxonomy source identifiers adopted for the challenge included the classification and nomenclature of all (and more) organisms found in the dataset. The “NCBI taxonomy resource dates 14-07-2017” archive could be downloaded from the sbv IMPROVER website and included the “nodes.dmp” and “names.dmp” files used to build the submission template available to the participants. A percentage of zero was assumed for all taxonomy IDs in the file submission template and not in the submission files. A participant’s submission was eligible for scoring if the participant had complied with the requirements described above, submitted predictions for all 19 samples, and included a write-up that described their methodology and computational tools used to solve the challenge.

### Scoring methodology

#### Gold standard

The submissions were scored by comparing the predictions to a gold standard that was unknown to the participants (Additional file 1). The gold standard corresponded to the true relative abundances (percentages of reads per species, genus, and phylum) of bacterial communities in the sample datasets or to the reported bacterial composition of the ZymoBIOMICS samples.

#### Procedure

To establish fair and meaningful performance scores, we used and aggregated complementary metrics. The scoring methods and metrics were reviewed and approved by an independent scoring review panel of experts before the closure of the challenge. To avoid optimization of models/methods for maximizing specific scoring metrics, the scoring methods and metrics were only disclosed once the scoring was completed, in accordance with the rules of the challenge. The participants’ submissions were anonymized before scoring. The scoring review panel reviewed the results of the scoring and approved the final team ranking. The three teams with the highest scores (and lowest ranks) were announced as the best-performing teams.

#### Metrics

Both qualitative and quantitative measures were used for scoring the participants’ submissions as well as for the extended benchmarking analysis. The OPAL software was used to compute scores for the respective metrics for the challenge scoring [28]. To have more flexibility with data formats for the extended post-challenge benchmarking analysis, we used our own code to compute the metrics. To enable fair comparison of the profilers, we implemented a unique reference taxonomy tree onto which taxa from profiler predictions were mapped (details in the “*Reference taxonomy tree for fair comparison of predicted taxonomy profiles among taxonomic metagenome profilers*” section).

The participants were asked to provide their submissions in form of relative abundances with the sum of all abundances in each profile equal to one. All the predictions obtained during the extended benchmarking analysis were normalized the same way. These normalized values were used for metrics calculation.

The F1 binary classification metric, which combines precision (purity) and recall (completeness), assessed how well—relative to the gold standard—a method detected the presence or absence of an organism at a specific taxon level. Confusion matrices were constructed for each prediction, and precision and recall were calculated as follows:

$$Precision=\frac{TP}{TP+ FP}$$

$$Recall=\frac{TP}{TP+ FN}$$

where *TP* is the number of true positives; *FP* is the number of false positives; and *FN* is the number of false negative species counts for the specific prediction and gold standard.

Precision and recall were used to calculate the F1 score as follows:

$$\mathrm{F}1=2\times \frac{P\times R}{P+R}$$

In case the analysis by sample failed for any reason, precision, recall, and the F1 score were automatically set to 0 (worst score).

The abundance metrics, L1 norm [15] and weighted UniFrac [29, 30], enabled assessment of how well a particular method reconstructed the relative abundances in comparison with the gold standard.

L1 norm is a measure of distance between the true and predicted abundances at a specific taxonomic rank and thus varies from 0 (a perfect match between the true and predicted abundances) to 2 (complete dissimilarity between the predicted and true abundances). The L1 norm was calculated as follows:

$$\mathrm{L}1=\sum_i^n\mid {x}_i-{x}_i^{GS}\mid$$

where *n* is the total number of taxa present in the gold standard; the prediction, *x*_*i*_ is the abundance of the i^th^ taxon in the predicted profile; and ${x}_i^{GS}$ is the abundance of the i^th^ taxon in the gold standard. In case the analysis by sample failed for any reason, the L1 norm was automatically set to 2 (worst score).

Weighted UniFrac is a tree-based taxonomy distance measure between the true and predicted abundances. Unlike the L1 norm, this metric enables consideration of the taxonomic similarities between the reported and true taxa. We used a taxonomy tree of seven ranks (superkingdom [or domain], phylum, class, order, family, genus, and species). We reported the results at the phylum, genus, and species levels for the challenge (use of the taxonomy tree implemented in the OPAL software) and only at the species level for the extended benchmarking analysis (reference taxonomy tree construction detailed in section “*Reference taxonomy tree for fair comparison of predicted taxonomy profiles among taxonomic metagenome profilers*”). The weighted UniFrac distance between two profiles was calculated as follows:

$$Weighted\ UniFrac\ distance=\frac{\sum_i^n\mid {a}_i-{b}_i\mid }{\sum_i^n{d}_i\ast \left({a}_i+{b}_i\right)}$$

where *n* is the total number of branches in the tree, *a*_*i*_ is the proportion of all reads descending from the i^th^ branch in the first community, *b*_*i*_ is the proportion of all reads descending from the i^th^ branch in the second community, and *d*_*i*_ is the distance corresponding to the number of branches from the tree root to the i^th^ branch (i.e., the weighted UniFrac distance ranges from 0 in case of a perfect match of two abundance profiles to 1 in case of complete dissimilarity). In case the analysis by sample failed for any reason, the weighted UniFrac distance was automatically set to 1 (worst score).

#### Randomness evaluation

To assess how far the predictions were from randomness, the participants’ prediction scores were compared with the distributions of random prediction scores. To generate 10,000 random predictions, N species were sampled (uniform distribution) from the 1886 reference species and randomly assigned a value between 0 and 1. For each species, the value was converted to a relative abundance by dividing it by the sum over all species and multiplying it by 100. The relative abundance for higher levels of taxonomy was calculated by summing the abundance values of all child taxa. For each metric and taxonomy rank, a distribution of random prediction scores was generated by computing the scores for 10,000 random predictions. A participant prediction was deemed to be better than random when its score was greater than the score of the 95th percentile (threshold) of the random prediction score distribution. All participant prediction scores falling below the 95th-percentile threshold score were replaced by the threshold score to give the same weight to all insignificant predictions by the participants.

#### Aggregation and final ranking

F1, L1 norm, and weighted UniFrac scores were computed for the predictions submitted for the 19 samples and 3 taxonomic levels (species, genus, and phylum), resulting in a total of 171 scores. For each sample, taxonomy level, and metric combination, the participant predictions were ranked by their respective scores. For the F1 score, where 1 was the best score and 0 the worst, the participants were ranked in decreasing order of scores. For the L1 norm and weighted UniFrac scores, where 0 indicates identity, the participant predictions were ranked in increasing order of scores. In the case of a tie, the average rank of the tied scores was assigned.

Aggregated scores consisted of the weighted sum of ranks of each sample–taxa–metric rank per team. The weight of the F1 score ranks was set to 1. The weight of the L1 norm and weighted UniFrac score ranks was set to 0.5 to account for the imbalance between abundance metrics and the binary classification metric. The weight of the score ranks for samples from the four experimental ZymoBIOMICS Microbial Community DNA Standards was further divided by four to account for the number of replicates. The best performer was the team with the lowest aggregated rank.

### Extended benchmarking of taxonomic metagenome profiling pipelines

#### Benchmarking datasets

In addition to the 19 challenge sample datasets, 85 publicly available metagenomic sample datasets with known composition were used to investigate the key steps in the challenge-winning pipeline and to benchmark a larger set of metagenomic profilers (Fig. 3 and Additional file 2, Sheet B). The selection criteria included the availability of data and gold standards, representation of a variety of microbiome samples from varying environmental settings and human/mouse organs, and use of simulated and real sequencing datasets. Additional file 2, Sheet B, provides detailed information on all sample datasets. To limit the over-representation of some datasets with highly similar composition and subsequent biasing of the results, all benchmarking sample datasets were split into 19 groups, each containing 1–64 samples, depending on the group (Fig. 3). All results for a group were calculated as the mean across the results for all datasets included in the group. Of note, the CAMI High (five samples) and CAMI Toy (64 samples) datasets were not used for evaluating the key factors affecting the performance of the top three Kraken-based pipelines.

#### Evaluation of key factors in the performance of the top three kraken-based pipelines

The three highest-performing pipelines used the Kraken tool, version 1.0, but differed in the combinations of key factors used, such as read filtering, reference databases, and abundance estimation with Bracken (Table 1). To better understand the difference in performance between these three pipelines, we investigated the impact of the key factors in various sets of combinations (Fig. 4 [Panel A] and Additional file 2, Sheet C). We expanded the analysis using additional benchmarking metagenomics datasets to ensure that the results and conclusions from the challenge test dataset were generalizable (Fig. 3 and Additional file 2, Sheet B).

#### Impact of read filtering

When applied, read filtering was performed by using Trimmomatic (version 0.38). The exact parameters of the tool varied by dataset (Additional file 2, Sheet H).

#### Impact of reference databases

Three reference databases were used to evaluate the key steps in the challenge-winning pipeline (Additional file 2, Sheet C). The first reference database, Microbial Challenge Bacterial, was constructed at the time of the challenge (July 2017) and included only NCBI bacterial sequences. The second database, Microbial Challenge Full, was also created at the time of the challenge and contained NCBI bacterial, archaeal, and viral sequences. The third database, Latest Bacterial, was constructed in April 2019 and contained NCBI bacterial sequences.

#### Impact of bracken

Bracken (version 1) results were always based on the output of the Kraken 1.1 version used for the challenge (Additional file 2, Sheet C). Bracken kmer database construction was performed with the *–read-length* parameter adjusted to the prefiltering read length in case of analysis with no filtering step. In case of filtering, the *–reads-length* parameter was adjusted to the postfiltering read length (see exact parameters in Additional file 2, Sheet H). Bracken analysis was performed by using the *-t* parameter equal to 10.

### Extended benchmarking taxonomy profiling pipelines

#### Taxonomic metagenome profilers

We expanded the benchmarking analysis to a total of 21 taxonomic metagenome profilers (Fig. 5 and Additional file 2, Sheet E), including different versions of tools such as Kraken with and without Bracken, CLARK or CLARK-S, mOTU, and MetaPhlAn as well as tools such as Kaiju, MetaPhyler, and Quikr. To avoid uneven representation of sample types, all taxonomic metagenome profilers were applied to all 104 benchmarking sample datasets aggregated into 19 groups for the results. Because Kraken 0, Kraken unique, Kraken 1.0.14, and Kraken 1.1 produced identical results for all benchmarking datasets, we decided to retain only the results for Kraken 1.1 to avoid redundant results. Additional file 2, Sheet E, lists the complete information on the taxonomic profiler version, reference database, and parameters used when running each taxonomic profiler. The analysis focused on species-level classification because it is at this taxonomic rank that profiler pipelines exhibit greater variations in performance in comparison with other taxonomic ranks.

#### Reference taxonomy tree for fair comparison of predicted taxonomy profiles among taxonomic metagenome profilers

The benchmarking datasets had been generated at various points in time, and most of the tested metagenomic profilers include a reference database that is generally precomputed. Therefore, to align the gold standards in terms of taxonomy and fair comparison of metagenomic profilers, we created a reference NCBI-based taxonomy tree and processed the output-predicted taxonomy profiles and the gold standards using this tree (Additional file 5). The reference taxonomy tree was generated from the NCBI complete database from October 29th, 2019. It was organized as a dictionary where the key is a species or subspecies taxID, and the value is an array containing the taxIDs of the corresponding species, genus, family, order, class, phylum, and superkingdom/domain (Additional file 4). The subspecies keys have the same value array as the species, and, thus, species is the lowest taxonomy level in the tree. Tree leaves for which either species or superkingdom was missing were removed. Leaves were also removed if more than three taxonomy levels were missing. If three or fewer taxonomic levels in the array were missing, they were replaced with the “unknown” taxID (Additional file 4). The tree structure generated contained 1,893,923 keys. Among these, 66,231 (3.5%) keys were missing one, two, or three taxonomic levels in their associated array.

Translation of taxIDs involved mapping of the taxIDs associated with each predicted taxonomy profile to the taxIDs of the reference NCBI-based taxonomy tree. If the taxID for a given predicted profile was not found in the reference tree, it was retrieved from the NCBI taxonomy website (https://www.ncbi.nlm.nih.gov/taxonomy/). We created a mapping table listing obsolete taxIDs and their replacements. The taxIDs associated with the predicted taxonomy profiles were mapped to the taxIDs of the reference NCBI-based taxonomy tree by using the following rules: (i) subspecies were collapsed under the taxID of the species to which they belong, and their abundances were added to the species abundance; (ii) obsolete taxIDs were replaced with the current taxID analogs; and (iii) if an obsolete taxID was replaced by a current taxID analog already present in the predicted taxonomy profile, the abundances associated with both taxIDs were summed, and the current taxID was used to replace the obsolete one.

#### Impact of low-abundance species filtering and determination of adaptive context-dependent thresholds

We investigated the impact of filtering out predicted low-abundance species on the overall performance of the taxonomic metagenome profiler pipelines. We initially used a 1% abundance threshold and then optimized this threshold by investigating the relationship between species abundances and within-sample diversities by calculating the Shannon index and identifying empirical thresholds for optimizing the tradeoff between false-positive and false-negative species across datasets. The Shannon dissimilarity index (H′) was calculated as follows:

$${H}^{\prime }=\sum_{i=1}^R{p}_i\ast \ln {p}_i$$

where *p*_*i*_ represents the relative abundance of the i^th^ taxa.

The Shannon index is a quantitative measure which reflects the diversity of species in a sample and thus accounts for the phylogenetic relationships among the individuals. We investigated how well the taxonomic metagenome profilers predicted the species diversity for each sample dataset in comparison with the diversity of the gold standards by calculating the coefficient of determination (R^2^) using the sklearn.metrics.r2_score package for Python.

## Supplementary Information

**Additional file 1.** 

**Additional file 2.** 

**Additional file 3.** 

**Additional file 4.** 

**Additional file 5.** 

**Additional file 6.** 

**Additional file 7.** 

**Additional file 8.** 

**Additional file 9.** 

**Additional file 10.** 

**Additional file 11.** 

## Data Availability

The raw metagenomics sequencing dataset generated for the challenge is available in the National Center for Biotechnology Information (NCBI) repository under NCBI BioProject ID PRJNA669653. All data generated (gold standard) or analyzed during this study are included in current article and its additional files.

## Abbreviations

sbv IMPROVER: Systems biology verification for Industrial Methodology Process Verification
CAMI: Critical Assessment of Metagenome Interpretation
HMP: Human Microbiome Project
wsr: Weighted sum of ranks
