# Supplementary figures and images for: Crowdsourced benchmarking of taxonomic metagenome profilers: lessons learned from the sbv IMPROVER Microbiomics challenge
Source: BMC Genomics. 2022 Aug 30;23:624. doi: 10.1186/s12864-022-08803-2 (PMC9429340; doi:10.1186/s12864-022-08803-2)

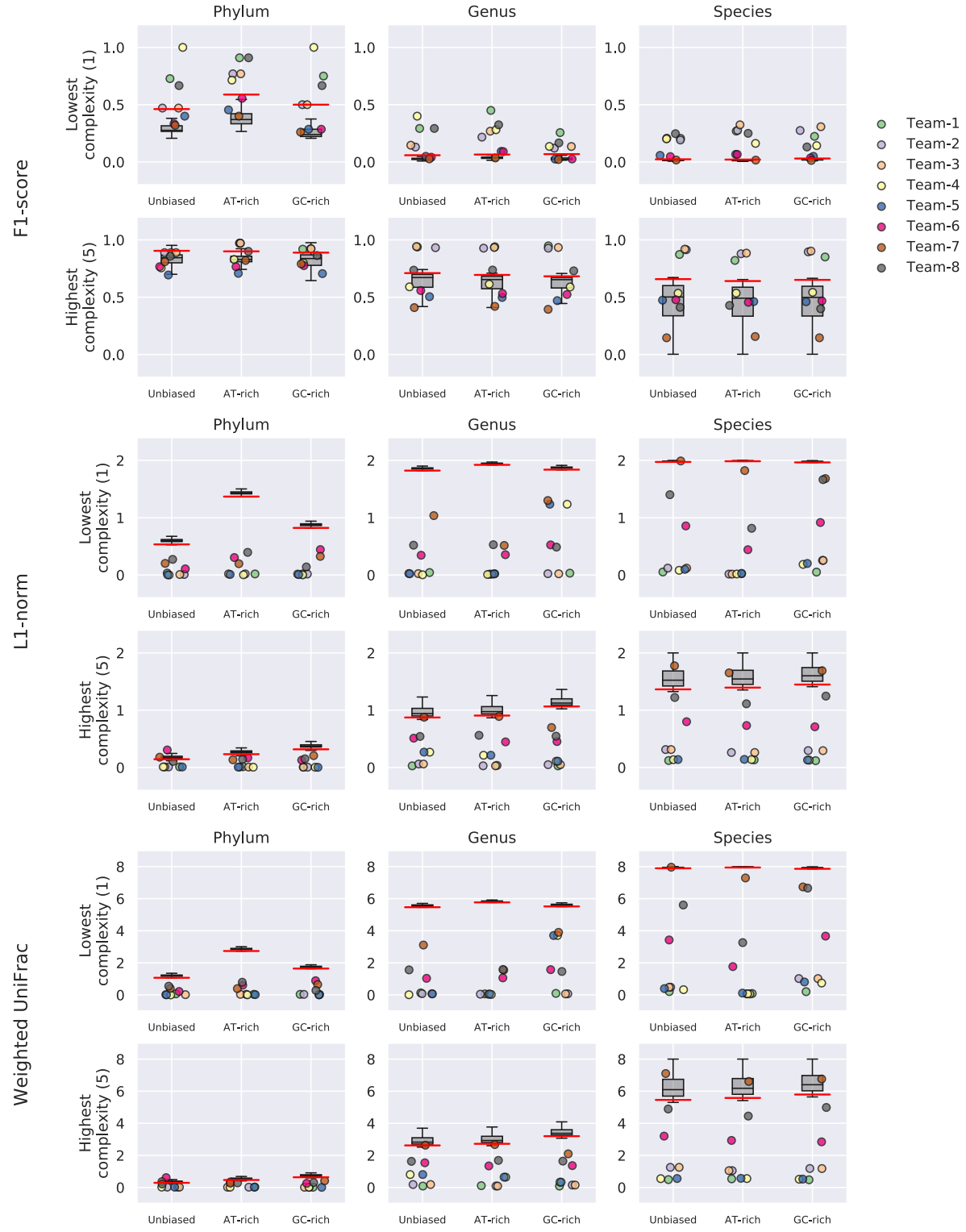

Supplement: Supplementary file 3 — Additional file 3. [file 12864_2022_8803_MOESM3_ESM.pdf]

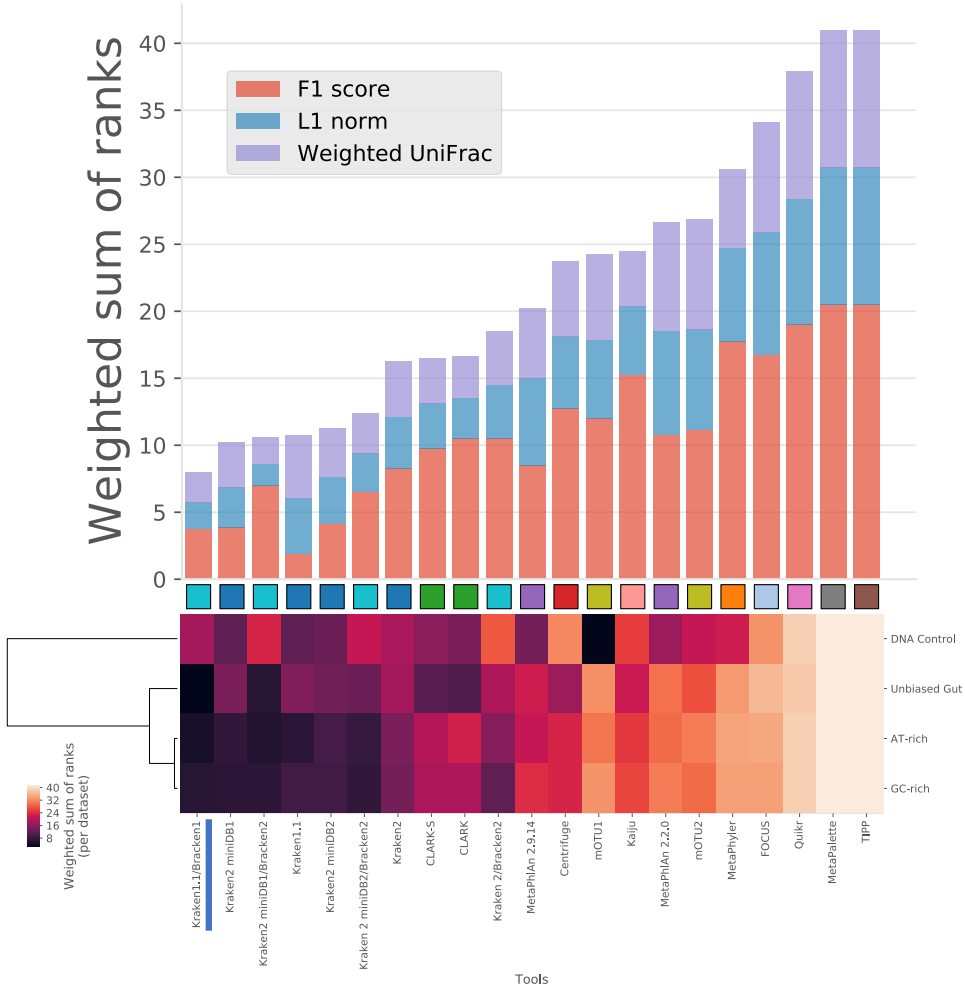

Supplement: Supplementary file 6 — Additional file 6. [file 12864_2022_8803_MOESM6_ESM.pdf]

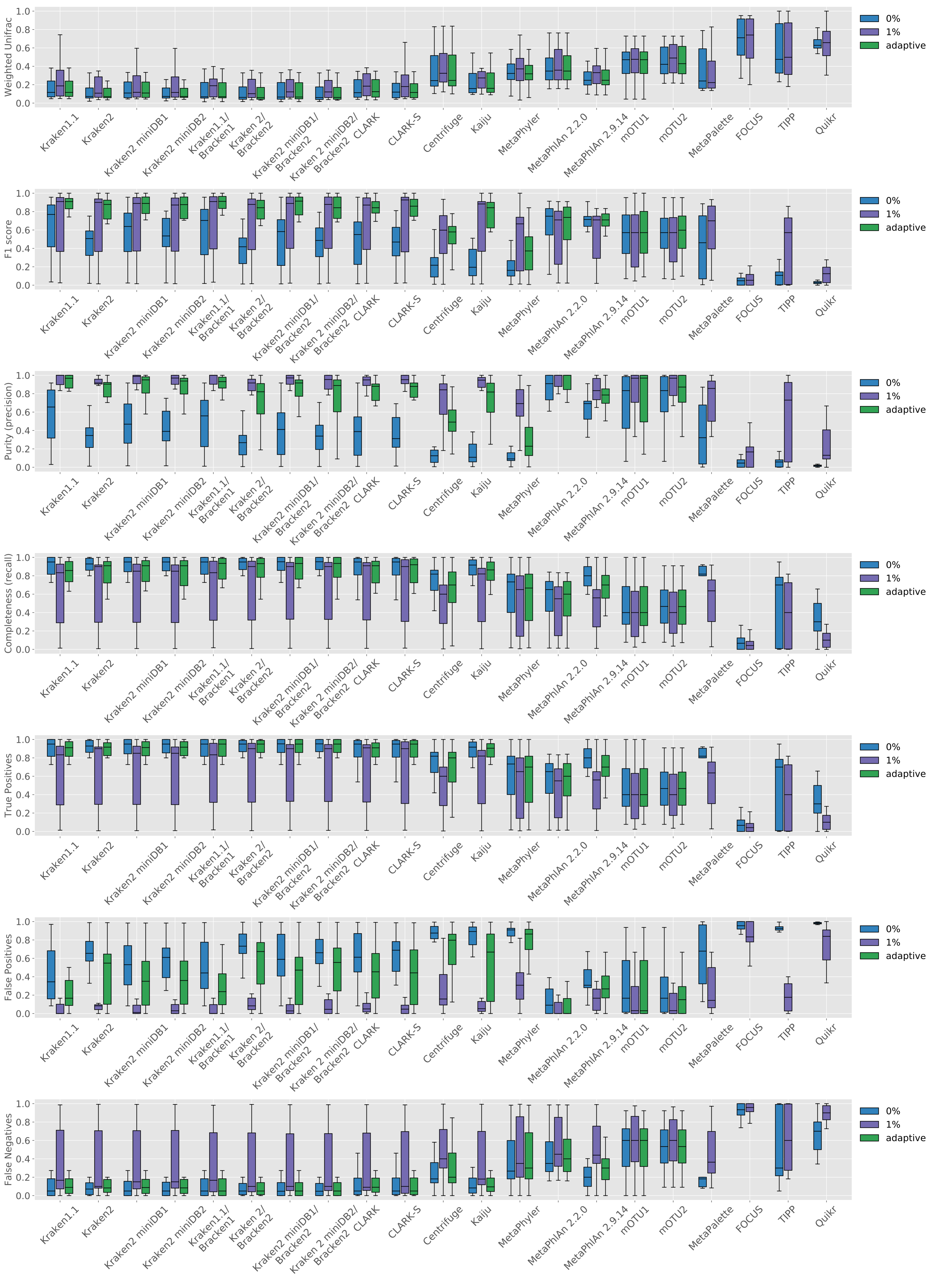

Supplement: Supplementary file 7 — Additional file 7. [file 12864_2022_8803_MOESM7_ESM.pdf]

# All Samples

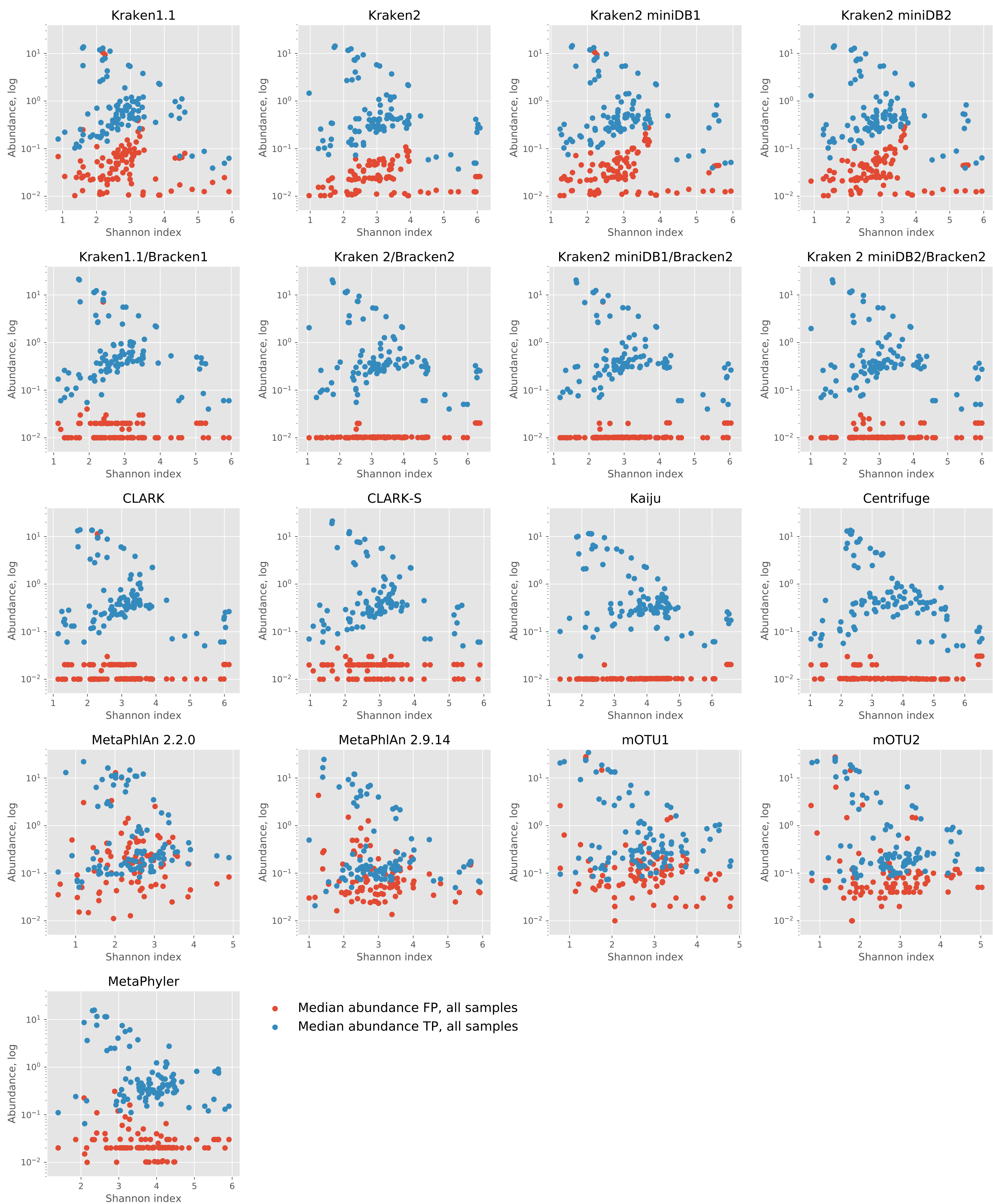

# Non - gut samples

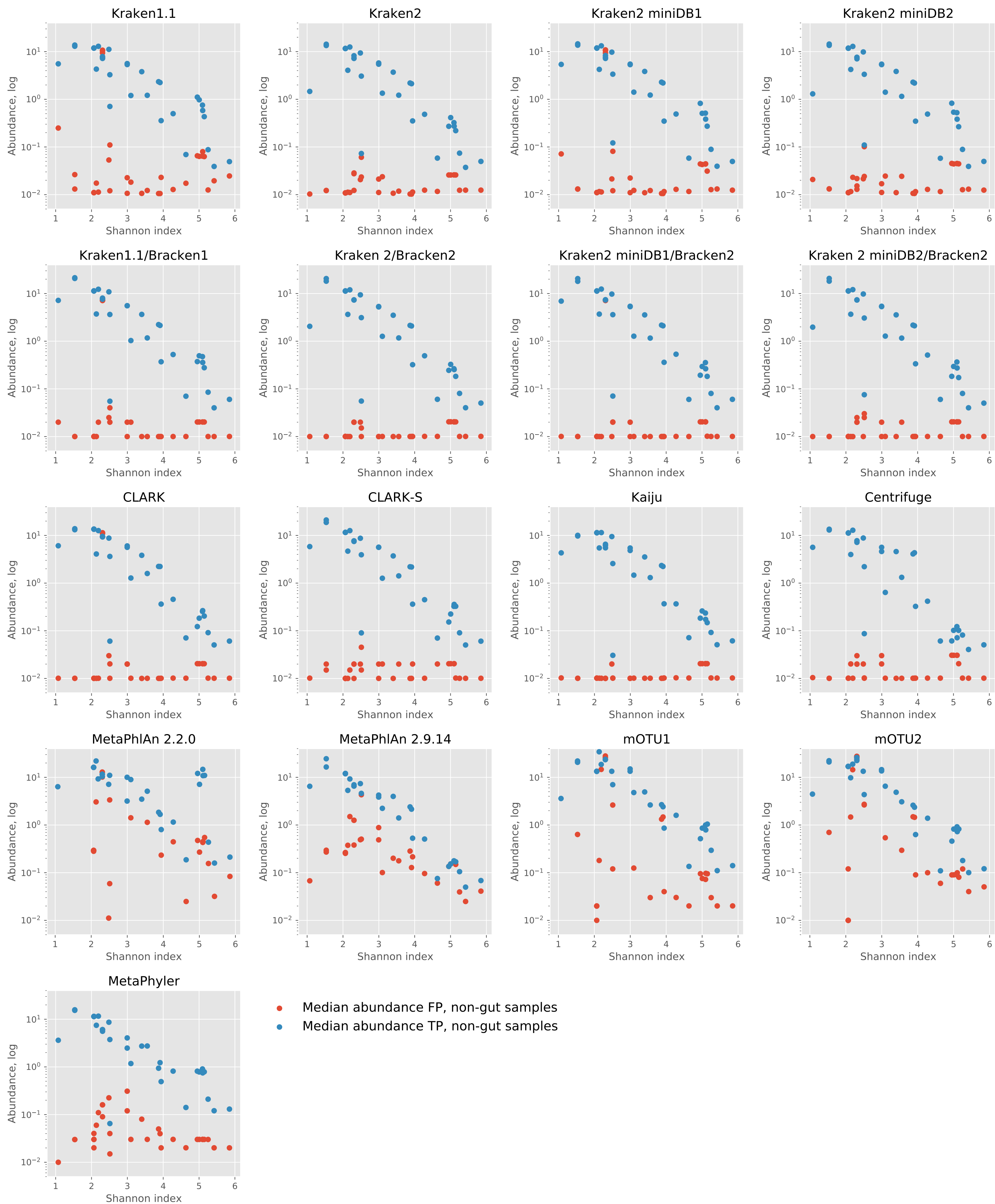

# G u t   S a m p l e s

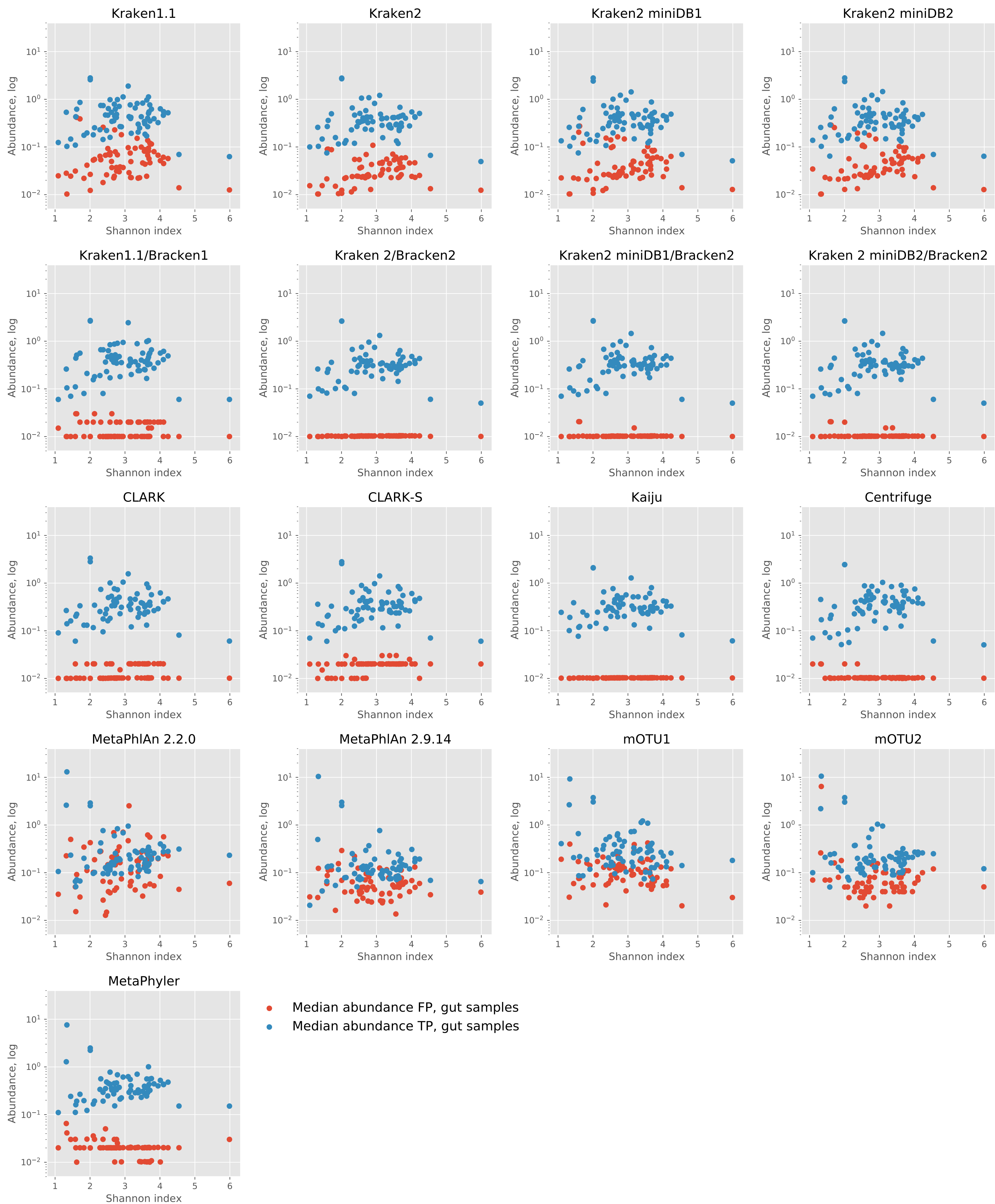

Supplement: Supplementary file 8 — Additional file 8. [file 12864_2022_8803_MOESM8_ESM.pdf]

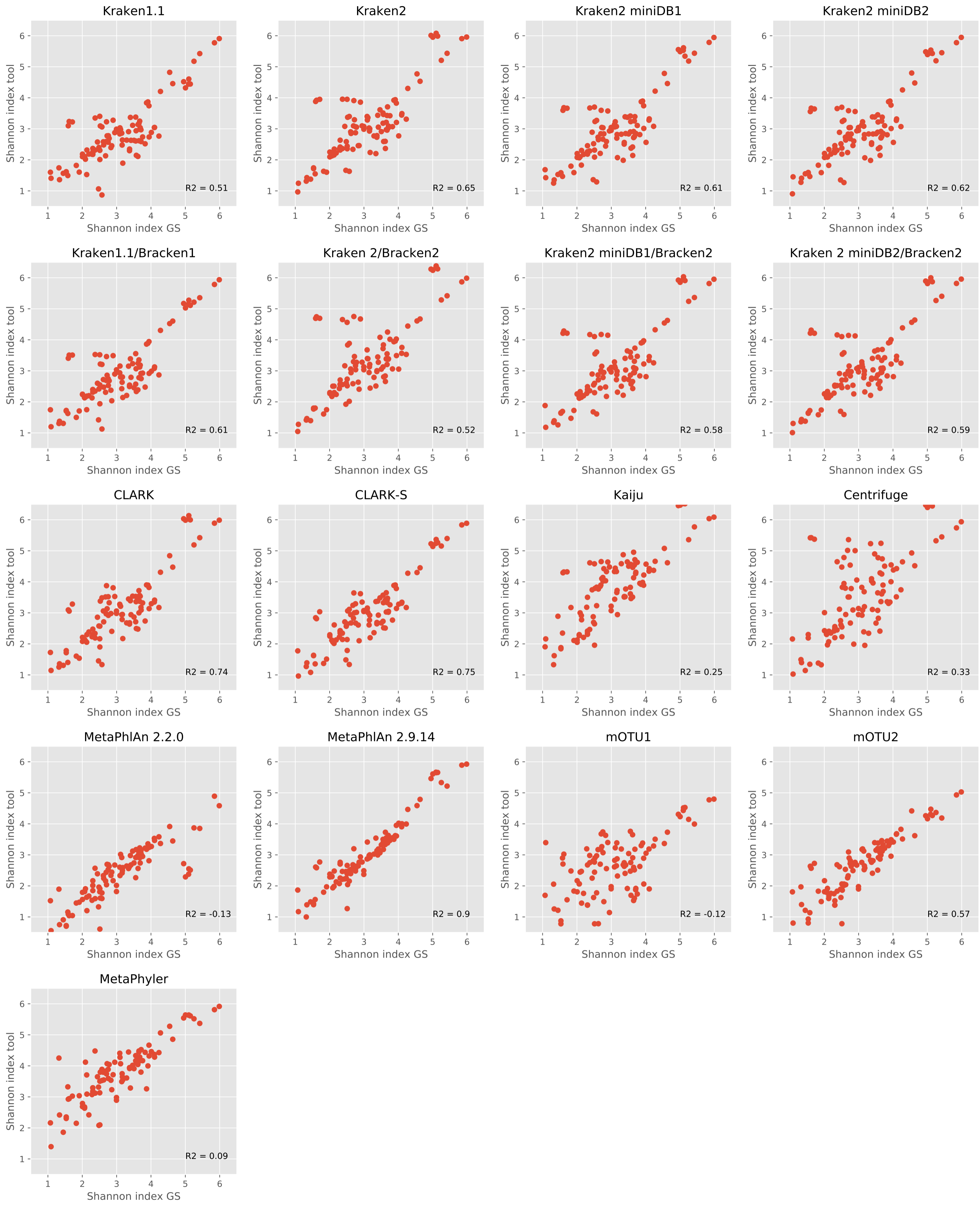

Supplement: Supplementary file 9 — Additional file 9. [file 12864_2022_8803_MOESM9_ESM.pdf]

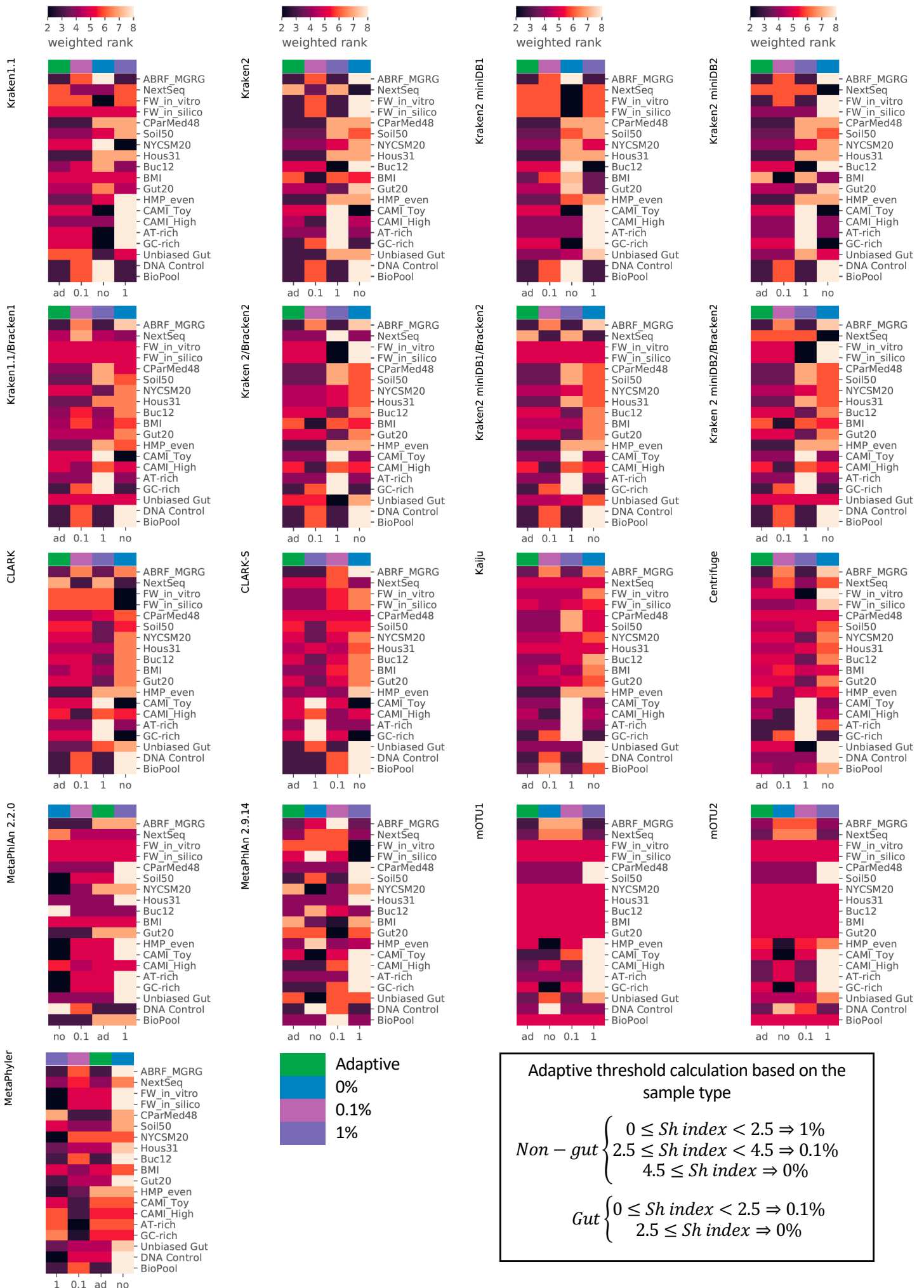

Supplement: Supplementary file 10 — Additional file 10. [file 12864_2022_8803_MOESM10_ESM.pdf]
