# Supplementary material for: Crowdsourced benchmarking of taxonomic metagenome profilers: lessons learned from the sbv IMPROVER Microbiomics challenge
Source: BMC Genomics. 2022 Aug 30;23:624. doi: 10.1186/s12864-022-08803-2 (PMC9429340; doi:10.1186/s12864-022-08803-2)
Supplement: Supplementary file 4 — Additional file 4. [file 12864_2022_8803_MOESM4_ESM.pdf]

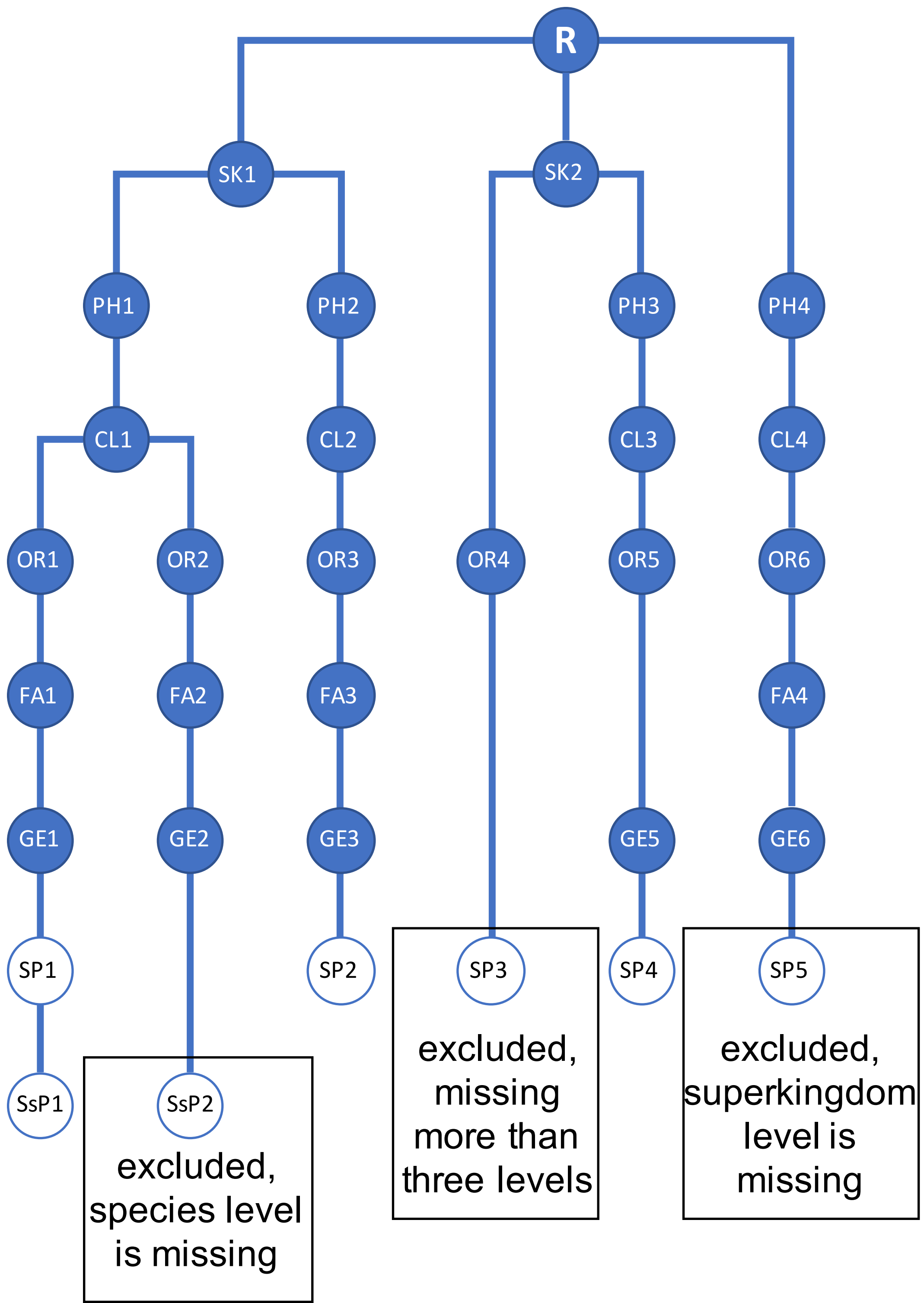

REFERENCE TREE ELEMENTS

keys	arrays						
**SP1**	**SP1**	**GE1**	**FA1**	**OR1**	**CL1**	**PH1**	**SK1**
**SsP1**	**SP1**	**GE1**	**FA1**	**OR1**	**CL1**	**PH1**	**SK1**
**SP2**	**SP2**	**GE3**	**FA3**	**OR3**	**CL2**	**PH2**	**SK1**
**SP4**	**SP4**	**GE5**	**FAu***	**OR5**	**CL3**	**PH3**	**SK2**

* - unknown

SsP: sub-species
SP: species
GE: genus
FA: family
OR: order
CL: class
PH: phylum
SK: superkingdom
R: root
