# Supplementary material for: Crowdsourced benchmarking of taxonomic metagenome profilers: lessons learned from the sbv IMPROVER Microbiomics challenge
Source: BMC Genomics. 2022 Aug 30;23:624. doi: 10.1186/s12864-022-08803-2 (PMC9429340; doi:10.1186/s12864-022-08803-2)
Supplement: Supplementary file 11 — Additional file 11. [file 12864_2022_8803_MOESM11_ESM.pdf]

For unbiased low and medium complexity (samples 19, 11, and 07)

- Random (uniform) selection of #species (with a variation of + 0% to 20% of #species) from the list of species identified in mouse gut microbiome of an internal mouse study
- Convert percentages to number of reads: the total number of reads (+ 0% to 10%) is 1, 5, 9*10^6 for samples 19, 11, and 07, respectively

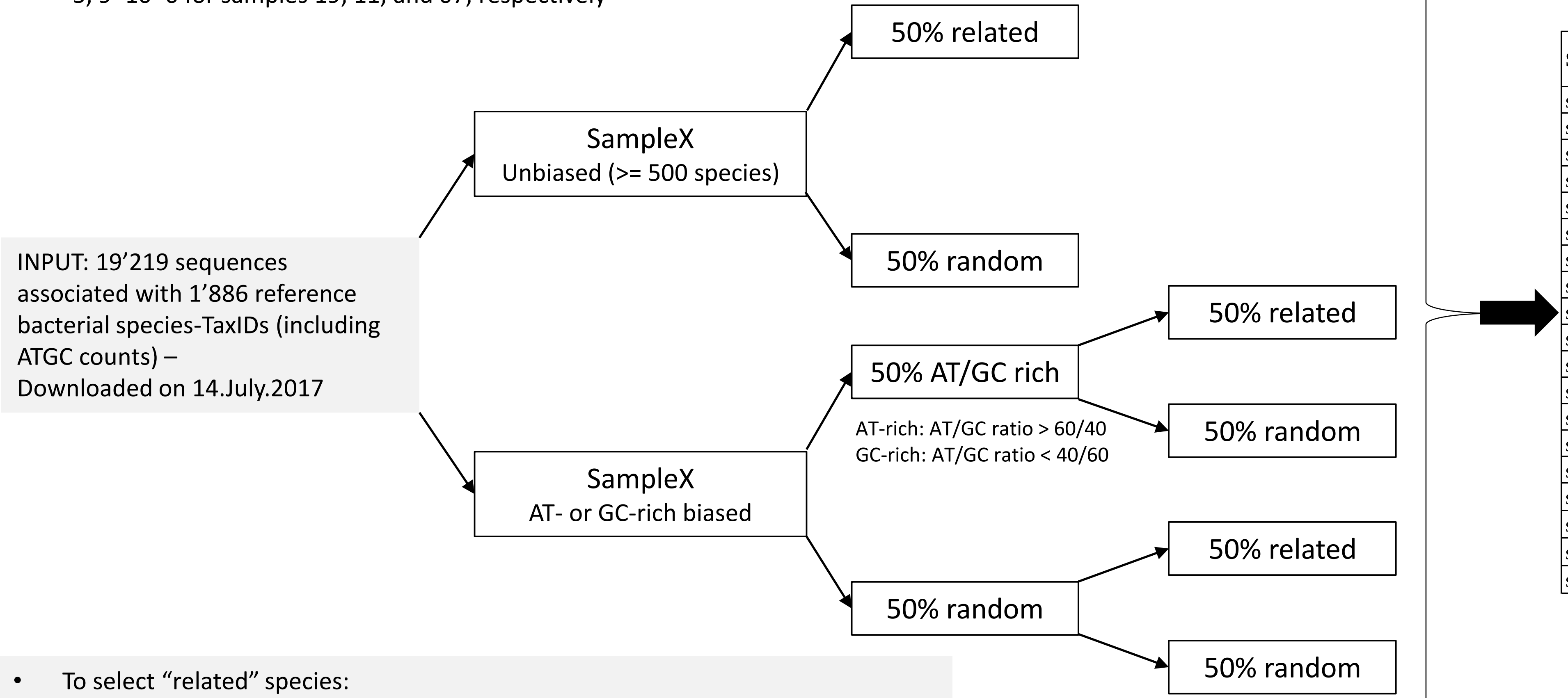

- To select “related” species:
  1. random (uniform) selection of genera including 4 to 11 species
  2. add all species from the selected genera (at least #species selected)

- To all selected species is assigned a random “coverage” number from a log-normal distribution of parameters:
  - mu= 1.2 and sd=0.8 for #species<500
  - mu= -1 and sd=1.0 otherwise

Sample	#Targeted species	#Selected species	Bias
sample19	10	12	No bias
sample18	10	14	AT-rich bias
sample08	10	19	GC-rich bias
sample11	40	42	No bias
sample05	40	52	AT-rich bias
sample06	40	46	GC-rich bias
sample07	120	132	No bias
sample13	120	124	AT-rich bias
sample15	120	124	GC-rich bias
sample10	500	536	No bias
sample01	500	508	AT-rich bias
sample04	500	528	GC-rich bias
sample14	1000	949	No bias
sample12	1000	912	AT-rich bias
sample03	1000	934	GC-rich bias
sample16	10	NA	No bias
sample17	10	NA	No bias
sample02	10	NA	No bias
sample09	10	NA	No bias
